# The Cardiovascular Risk Awareness and Health Lifestyle of Italian Women

**DOI:** 10.3390/jcm13113253

**Published:** 2024-05-31

**Authors:** Adele Lillo, Ettore Antoncecchi, Valeria Antoncecchi, Giovanni Battista Zito, on behalf of the A.R.C.A. CARIN WOMEN Survey Investigators

**Affiliations:** 1 Outpatient Cardiology Ospedale “Fallacara” Triggiano, 70019 Triggiano (BA), Italy; valeriaantoncecchi@libero.it; 2 Poliambulatorio Medigea, 70026 Modugno (BA), Italy; cecchiettore@outlook.it; 3 Cardiology Service, Local Health Unit (LHU) Naples 3 South, Associazioni Regionali Cardiologi Ambulatoriali (A.R.C.A.), 80045 Rome, Italy; giovannizito2013@gmail.com

**Keywords:** cardiovascular risk factors, women, gender medicine, cardiovascular prevention, awareness

## Abstract

**Background:** Cardiovascular (CV) disease is the leading cause of death in women, but few of them are aware of the CV risks (CVRs). Most women are not aware of all the CV risk factorsand their knowledge often still does not improve their lifestyle. **Methods:** The Carin Women is a survey conducted among Italian women by filling out a questionnaire in the waiting rooms of clinics. The aim was to determine the level of awareness of women’s cardiovascular risk, knowledge of risk factors, and lifestyle. A total of 5590 questionnaires were completed in two different periods. **Results:** Median age was 56 (IQR 46–65); BMI was 25 (IQR 22–29). Schooling, marital status, and rate of risk conditions were assessed; 311 women (5.57%) had already suffered a cardiovascular event. The relationship between the CV events and the number of traditional risk factors was significant. A similar curve, but without significant differences, was reported for non-traditional risk factors. From the total number of women, 23% with a high CVR and 62% with a very high CVR underestimated thei risk regardless of their level of education. Up to 43% of women underestimated female CV risk compared to male risk. Women showed a good knowledge of traditional risk factors, but only a few of them had a healthy lifestyle: 21.86% were smokers, only 45.88% performed sufficient physical activity, 27.55% did not recognize they were overweight, and only 30.4% consumed more than two daily portions of fruit and vegetables. Most women (86%) need more information about CVR. **Conclusions:** Italian women underestimate female CVRs and their own CVR.

## 1. Introduction

Cardiovascular diseases (CVDs) are the leading cause of mortality in women globally. It is estimated that in 2019 alone, there were 275.2 million patients diagnosed with CVD and 8.94 million died from it [1]. This bitter record is still unknown today, or at least underestimated, even by various health professionals and even more so by the majority of the female population who continue to perceive oncological pathology as the main threat to their health [2]. Although decades of campaigning have helped raise awareness of the impact of CVDs on women, they remain under-studied, under-recognized, under-diagnosed, and under-treated [1,3].

Previous studies have shown, especially in the United States and Australia, that there is a lack of knowledge that CVDs are the leading cause of female death, that they are not a predominantly male problem, and that women underestimate their cardiovascular risk. In Europe, few studies about the prevalence of cardiovascular risk factors (CVRFs) and female risk awareness have been conducted on a large population [4], and only one Italian study [5] was published while we were completing the present work.

While it is beginning to be known that traditional risk factors (TRFs) do not act equally in both sexes [2,6,7,8,9], not much is known about the prevalence of new non-traditional CV risk factors (NTRFs). NTRFs, also called “emergent” risk factors affecting females, can be non-sex-specific or sex-specific. The former are not exclusive but predominant in the female sex (autoimmune and chronic inflammatory diseases, damage related to breast cancer therapies), probably supported by a greater effect on immunity and inflammation by estrogens [10,11]; the latter NTRFs are exclusive to women: some of them are related to hormonal status (age of menarche and menopause, polycystic ovary syndrome), others are related to pregnancy (hypertension and gestational diabetes, repeated miscarriages and premature births). Further CVRFs, often cited but not well characterized by clinical studies, involve the female sex more frequently for sociocultural reasons: anxiety, depression, chronic stress, and others that are more difficult to assess such as cultural and socioeconomic level, environmental conditions, and situations of domestic violence. All these latter factors can influence CVR by both direct and indirect mechanisms, favoring unhealthy behaviors (e.g., sedentary lifestyle, smoking, alcohol abuse).

CVDs in women are underestimated not only by women themselves but also by healthcare professionals [12], among whom the prejudice that women have a lower CVR due to estrogen production in childbearing age still carries a strong weight, generating false optimism about their level of protection. There is a gap in knowledge and awareness about female risk among healthcare professionals which is even more evident among male physicians [13], resulting in under-treatment [14,15,16].

The prevalence of non-traditional risk factors is unknown in Italian women, as well as the perception of female CVR compared to male CVR, the estimation of their CVR, and their need for further information. There is also little data on lifestyle. We believe that such knowledge can improve the prevention and treatment of CVDs in women.

## 2. Materials and Methods

The CArdiovascular Risk awareness of ItaliaN WOMEN (CARIN WOMEN) survey is a multicentre, nationwide study, conducted by the Medicine Gender group of the Italian association A.R.C.A. (Associazioni Regionali Cardiologi Ambulatoriali). We developed a questionnaire including demographic and clinical data, personal habits, and questions concerning the perception of cardiovascular risk factors. The questionnaire was submitted to women afferent to out-patient clinics, mainly cardiological, distributed throughout the Italian territory and filled out anonymously during their permanence in the waiting room. The originally planned duration of the enrolment of 6 months was reduced to 4 due to the COVID-19 pandemic in January–April 2020. We extended the questionnaire collection in a second period from January to June 2021. In the second period, we added some questions to the original questionnaire: the first form was reported in a previous paper [17] and the second form is shown in Appendix A. We collected 5590 questionnaires: 2586 are from the first period and 3004 from the second. A total of 49 hospitals and territorial outpatient clinics were involved in the north, centre, and south of Italy.

Data management and privacy.

A progressive identification number (not linked to the patient’s identity) was assigned to each questionnaire, together with an identification code of the investigator. The investigator’s medical specialty and the city where he/she worked were requested. All the questionnaires were collected, and data were entered into a web-based central database by a single operator.

Endpoints.

The main endpoints were

- to evaluate Italian women’s awareness of cardiovascular risk (both risk of women compared to risk of men and own risk);
- to evaluate the knowledge of traditional cardiovascular risk factors and their impact on cardiovascular events.

The secondary endpoints were:

- to evaluate the prevalence and distribution of traditional and non-traditional risk factors including gender-specific cardiovascular risk factors;
- to collect information about lifestyle habits: Smoking, diet, and physical activity;
- to know the need for receiving further information on CVR.

Inclusion and exclusion criteria.

Inclusion criteria: every woman in the waiting room who wanted to fill out the questionnaire anonymously. No exclusion criteria for entering the study were contemplated.

Statistical analysis

Continuous variables are expressed as mean ± SD (standard deviation). The normal distribution was assessed using the Shapiro–Wilk test. If the distribution was not normal, we reported data as median and Interquartile Range (IQR). Comparisons between groups were performed using Student’s *t*-test for continuous variables with normal distribution or Mann–Whitney’s U test for non-normal distribution. Categorical variables are expressed as numbers and percentages; we used the Chi-square and Fisher’s exact tests for comparison. For all comparisons, a *p*-value < 0.05 was considered statistically significant.

## 3. Results

### 3.1. Demographic and Anthropometric Characteristics of the Population

The characteristics of the 5590 women who participated in the survey are reported in Table 1. The median age was 56 (IQR 44–65). Based on age, participants were divided into 4 subgroups: <46 (21.14%); between 46 and 55 (26.44%); between 56 and 70 (37.07%); and >70 (14.19%). The median Body Mass Index (BMI) for 95.9% of the patients who reported weight and height was 25 (IQR 22–29). Three BMI groups were distinguished: normal (53.11%), overweight (30.61%), and obese (16.28%) women.

Furthermore, four conditions regarding marital status (single, married, separated or divorced, widowed) and four levels of schooling (primary, lower middle, upper middle, and university) were considered. The data show that 35% of women did not reach a high school diploma and about 13% did not answer this question; only 17% were graduates. The percentage of women with only primary school licenses was 12%.

### 3.2. Prevalence of Cardiovascular Risk Factors

The risk factors investigated were divided into traditional (TRFs) and non-traditional (NTRFs). TRFs included arterial hypertension, diabetes mellitus, hypercholesterolemia, and smoking habits. NTRFs concerned both gender-specific (pregnancy-related RFs: preterm births, hypertension, diabetes in pregnancy, repeated miscarriages) and non-gender-specific RFs, represented by autoimmune diseases, chemo- or radiotherapy treatments for breast cancer, anxiety, and depression. Sixty-nine percent of the women had at least one CVRF, 56% had at least one TRF, and 39% had at least one NTRF. BMI, age, and menopause are important CVRFs not listed in the abovementioned groups.

The most frequent CVRF declared was arterial hypertension (31.72%), followed by cigarette smoking (21.68%), hypercholesterolemia (17.66%), and diabetes mellitus (10.04%); obesity or overweight was perceived in 20.23%, and this percentage was quite different from the 40.83% we determined by BMI.

Among NTRFs the most frequent was represented by autoimmune diseases (15.15%), immediately followed by breast cancer treatments (15.13%) (Table 2).

Anxiety, depression, and menopause were investigated only in the second form in 3004 women (Table 3): the prevalence of anxiety without depression was 15.85%, the prevalence of depression alone was 2.60%, and the prevalence of both together was 5.66%; 24.15% of the women confirmed at least one of the two. Women not in menopause were 27.8%; 3.06% had been in menopause for less than a year and 3.10% for less than 2 years; 61.92% had been for more than two years. The women who did not answer were 4.13%. Forty-six women out of 3004 (3.7%) over 60 years stated that they were not in menopause.

### 3.3. Prevalence of Cardiovascular Diseases

Among overt cardiovascular diseases (CVDs), the most frequently reported event was heart failure (2.99%), followed by stroke or TIA (1.74%) and myocardial infarction (1.57%) (Table 3).

**Figure 1:**
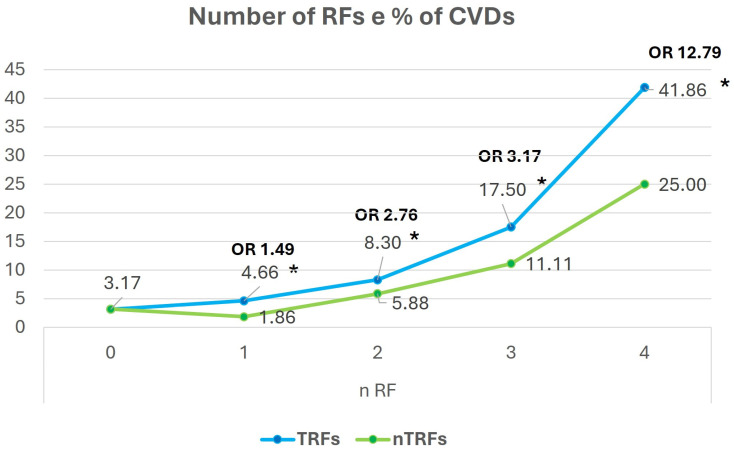
Prevalence of CVDs compared to the number of CVRFs. CVDs, CVRFs, TRFs, and NTRFs: see text. OR: Odds Ratio. * Significance in comparison with the FR 0 group.

**Figure 2:**
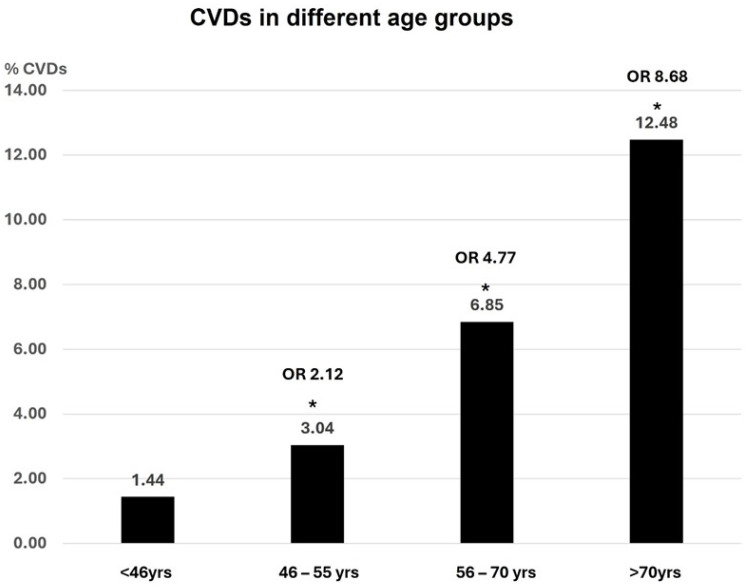
Prevalence of CVDs according to age. (OR: Odds Ratio; yrs: years; CVDs: cardiovascular diseases). * Statistically significant in comparison with the age group <46 years.

As expected, the prevalence of CVDs increased with the number of TRFs and NTRFs and with age, which is itself a powerful CVRF (Figure 1 and Figure 2). While the increased percentage of CVDs was statistically significant for TRFs, it was not significant for NTRFs, although the trend of the two curves is similar.

The presence of two to four TRFs significantly increased the prevalence of CVDs from 3.17 (0 RFs) to 41.86% with an OR of 12.79 (four RFs). The presence of two to four NTRFs caused a similar exponential curve of the CVDs prevalence without a statistically significant increase: this may be due to a low number of events (Figure 1). The CVD prevalence increased significantly in each age group, by approximately 12 times in the over 70 yrs age group, as compared to the under 46 yrs age group, with an OR of 8.7 (Figure 2).

### 3.4. Awareness of One’s Own Cardiovascular Risk

This topic was investigated only in 3004 pts (second form).

Regarding the awareness of their own cardiovascular risk, 30.83% of enrolled women answered that it was low, 48.70% that it was medium, and 10.85% that it was high.

Table 4 shows the opinions of groups of women with different TRFs numbers on their own CVR. In all the groups, 47% to 61% of the women thought they had a medium CVR. Interestingly, 23% of women with one or two CVRFs believed they had a low CVR, and only 38% of women with three to four CVRFs believed they had a high CVR. 

In assessing the relationship between the number of CVRFs and the perception of one’s own CVR according to marital status and schooling, we considered menopause as a CVRF in addition to TRFs. The percentage of women at increased CV risk was very high in all marital status groups (53 to 71% with one or two CVRFs and 8 to 25% with three to five CVRFs); the CVRFs number was lower in unmarried younger women; only a low percentage of women in all groups perceived that they had an increased CVR, and this underestimation was higher in the married women’s group (only 10.85% believed they had an elevated CVR) (Table 5 and Table 6).

The number of TRFs decreased as education increased, but in all levels of education, only a low percentage of women perceived their CVR increasing (Table 6).

A range from 40% to 53% of women in all educational grades believed they were at a medium CVR; the higher the school education, the higher the belief of a low CVR, the lower that of a high CVR, and the lower the underestimation: primary school, 31%, middle school, 17.5%, high school, 11.5% and university, 6.5%). Moreover, it was possible to find a statistically significant difference by comparing women with primary school vs. university education (believing own CVR as low respectively, 26% vs. 34%, *p* = 0.009 and as high as 15% vs. 9%, *p* = 0.006). 

**Figure 3:**
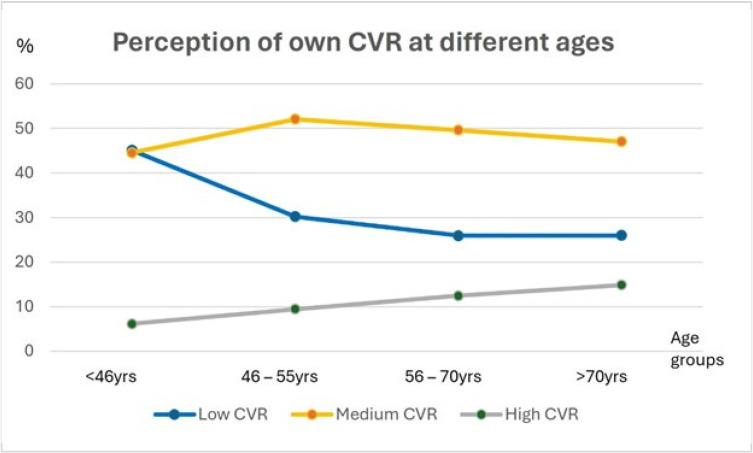
The graphic shows the perception of one’s own CVR in different age groups. Coloured lines indicate the trend in perception of own risk as low (blue), medium (yellow), and high (grey).

The menopausal women believed to have a high CVR more than women not yet in menopause (12.71% vs. 6.35%, *p* < 0.001) and were less likely to have a low CVR (26.01% vs. 42.28%, *p* < 0.001) (Table 6). The perception of a high own CVR was directly proportional and that of a low own CVR inversely proportional to age, with significant differences between the groups except for the between 56 and 70 yrs and over 70 yrs groups (Figure 3).

Regarding the item of own risk perception, it is quite interesting to observe the difference between the real prevalence of BMI > 26, which was 46.89% (Table 2), and the percentage of patients who declared to be overweight (Table 2), which was 20.23%. Out of 873 obese women and 1641 overweight women, only 511 (58.53%) and 463 (28.21%), respectively, affirmed to have this risk factor.

### 3.5. Awareness of Female versus Male CV Risk

We assessed the perception of cardiovascular risk of the interviewees by asking them if the risk of undergoing CV events for women, as compared to men, is lower, equal, or higher: 18.46% of 5090 women considered it lower, 38.75% equivalent, and 13.42% higher, while 17.87% did not know and 6.67% did not answer. Thus, 36.33% to 43% of women underestimate or do not know female CVR.

### 3.6. Knowledge of Traditional Risk Factors

The data show a good knowledge of the TRFs: 5173 (92.54%) participants were aware that arterial hypertension is an important cardiovascular risk factor, 205 (3.67%) denied it and 212 did not answer the question. Likewise, regarding diabetes mellitus, 4296 women (76.85%) were aware and 552 (9.87%) denied it; regarding hypercholesterolemia, the proportion was 5088 (91.04%) and 181 (3.24%), respectively; regarding obesity, 5061 (90.54%) and 84 (1.5%), respectively; and regarding cigarette smoking, 5259 (94.08%) and 175 (3.13%), respectively.

### 3.7. Lifestyle

Interestingly, 5259 women (94.08%) vs. 175 (3.13%) recognized smoking cigarettes as a CVRF (56 did not answer), but 1212 (21.68%) were active smokers. However, there were different levels of smoking: 804 smokers (66.56%) smoked up to 10 and 408 (33.44%) more than 10 cigarettes a day (Table 2).

Regarding physical activity, although about 89.82% of women recognized that moderate physical activity is useful in reducing the risk of cardiovascular events, only 13.36% of them exercised regularly, 34.53% walked regularly, 45.88% of women declared to be sedentary, and about 6% did not answer the question (Table 7).

We also explored eating habits, asking how many portions of fruits or vegetables they consumed every day: the majority (61.31%) said they consumed one or two portions, 30.4% more than two portions, 2.99% said they did not eat any at all and no answer by 5.3% (Table 7).

When asked which lifestyle goals are the most difficult to achieve, 1649 women (29.50%) indicated correct eating habits, 1890 (33.81%) exercising regularly, and 815 (14.58%) quitting smoking. For 818 women (14.63%), all the previous ones seemed hard to reach (Table 7).

### 3.8. Need for Information on Cardiovascular Risk

Most women (87.44%) claimed the need for more information on their CVR and how to reduce it versus 4.9% believing to know enough about it: 55.85% indicated that it should be the family doctor to provide them the correct information, 21.59% the cardiologist, only 8.01% wanted to obtain information from other sources such as the media, while 8.01% proposed multiple solutions.

## 4. Discussion

Women’s awareness that CVDs are the leading cause of female death is still not widespread today. Everyone considers cancer to be the most fearsome risk of death. In the literature, different papers confirmed this topic. In 2012, Lori Mosca et al. [18] demonstrated the low awareness of women in the U.S. in only 56% of white women and 33% of black and Hispanic women; the data were disappointing but showed an improving trend in three-year steps since 1997. In 2016, Ramachandran et al. [19] reported the poor ability of women to correlate CVRFs with CV events in a meta-analysis of 21 studies. In 2019, in contrast to the positive trend reported by Mosca et al. [18], Mary Cushman et al. [20] reported that awareness of CVDs as a leading cause of death had declined from 65 to 44 percent over the past 10 years, especially in non-white and younger women. Gooding HC et al. [21] showed an insufficient knowledge of CVD-related risk in a small group of 331 young women aged 15–24. Maffei S. et al. [5] confirmed the underestimation of CVD (69.8%) as the leading cause of death in 4454 Italian women. In the CARIN WOMEN questionnaire, we did not deal with this important problem that we have taken for granted, focusing mainly on the perception of female CVR compared to male CVR, on the awareness of one’s own CVR, and on the knowledge of TRFs; all these are the primary endpoints of our work.

### 4.1. Female versus Male Cardiovascular Risk

We know that it is hard to compare female to male CVR. As mentioned above, CVDs are the leading cause of women’s death in developed countries. Women have fewer CV events before menopause but have more CVDs and worse prognosis after. Therefore, it is not a tenable statement that the male CVR is superior to the female one, but many people think so. Data from our paper show that 25% of women did not answer and 18% considered female CVR lower. Only 57% of Italian women knew that female CVR was at least at the same level as the male risk. In the report by Maffei et al. [5], 60% of women considered CVDs as an almost exclusively male condition. Our result is consistent but slightly better (57% vs. 40% of women were aware of their CVR). This result was expected because the cultural environment, even that of health professionals, has until now considered CVR as something of the male sex with a consequent underestimation of symptoms and diagnoses, thus worsening prognosis in women.

### 4.2. Own Cardiovascular Risk Awareness

Another CARIN WOMEN main endpoint was to evaluate the awareness of one’s own CVR. The population investigated in this survey was predominantly in primary prevention (only 6.3% with previous CV events) and quite heterogeneous in age, schooling, and marital status. The question about the perception of one’s CVR was asked only in 3004 patients (second survey): 51.71% of women stated to have at least one TFR, but only 9.09% believed they had an increased CVR. Awareness of one’s CVR was low among those who should have it the most: in fact, only 37.76% of women with high CVR (three or more RFs) considered themselves as high-risk patients; 23.03% of patients with intermediate CVR (one or two RFs) considered herself at low CV risk. The higher the risk, the higher the underestimation, in contrast to Maffei’s report [5] and in accordance with JJ Monsuez et al. [22]. The latter showed that, in a group of 5240 women of high school level, the perception of one’s CVR was low for 1.279 (20.4%), moderate for 3.710 (63.3%), and high for 893 (16.3%) women against a Framingham score with low CVR in 40.8%, moderate in 25.2% and high in 33.8%. JM Kling et al. [23] conducted a study on a group of 294 women, 99% of whom recognized CVDs as the leading cause of death; of these, 84% were at high CV risk and 12% were at very high risk, but only 45% perceived that they were at risk (48% of high-risk women and 21% of very high-risk women did not perceive any risk). In 2019, the Berlin Female Risk Evaluation (BEFRI) study [4], one of the few European studies, showed that among 1066 women aged 25 to 74 years, 48.65% underestimated their CV risk; the extent of underestimation increased mainly with age (OR = 3.5 for age >50 years compared to <50 years) as well as with the association of other mostly socioeconomic conditions. The Virgo Study (VS) [24] published in 2015 was conducted on 3501 patients (2349 women and 1152 men), 85% from the USA and all with myocardial acute infarct. The questionnaire was about the perception of one’s risk before the event. Approximately 67% of women and 65% of men had three or more CVRFs, and 97% and 98.8% had at least one CVRF, respectively; only 52.2% of women and 55.8% of men perceived that they had an increased CVR. 

As pointed out in the BEFRI study, other socioeconomic and demographic factors also seem to influence the perception of one’s risk. One of the social factors, considered in the CARIN WOMEN survey, is marital status. In our survey, the perception of having an increased CVR was higher in widowed or separated/divorced women (17 and 15 percent) vs. married or single women (10 and 8 percent). This was justified for widows who have a higher mean age (71.65 years) and a higher number of CVRFs, and for single women who, on the contrary, have a lower age (44.92 years) and a lower number of CVRFs; it is not clear why separated or divorced women consider themselves to be at increased risk more than married women who are older (55.13 vs. 58.14 years, respectively) and with a higher number of CVRFs. Conversely, married women underestimated their CVR the most. Probably psychological rather than objective factors influence the perception of risk. Perhaps women who live alone have a more pessimistic or more self-centred outlook, and in contrast, those who live with a partner have more optimism or are less attentive to their problems by caring more for their partner or family. 

In the CARIN WOMEN survey, the perception of having a high CVR increased and that of having a low CVR decreased with increasing age, the latter especially between 46 and 55 years of age (the age of menopause). However, since CVR also increases with age, the underestimation of one’s CVR increases, as reported by the BEFRI study [4] but in contrast to the Italian IGENDA study [5].

Level of schooling, which is an important socio-cultural factor, also seems to have a significant impact on effective risk, as the number of CVRFs decreases with increasing education. Considering self-perception, underestimation of one’s risk was across the board in all levels of schooling, where only about 13 to 15 percent of women recognized that they had an increased CVR, but 56 to 72 percent had a high CVR and 12 to 24 percent had a very high one. In any case, own CVR underestimation decreased with the level of schooling.

Another demonstration of how much one’s own risk is underestimated is the perception of one being overweight: 46.89% had a BMI >26 but only 20.23% reported being overweight. Regarding BMI, 71.79% of women with a BMI of 26–30 and 41.47% of women with a BMI > 30 did not recognize that they were overweight. 

### 4.3. Knowledge of Traditional Risk Factors

The underestimation of one’s own CVR compared with the excellent knowledge of TRFs (91–94% of respondents) was slightly reduced only for diabetes mellitus (76%), confirming the data on Italian women reported by Maffei et al. [5].

### 4.4. Prevalence of Non-Traditional Risk Factors

About 15 percent of the women surveyed said they had a chronic autoimmune or inflammatory disease, 15 percent said they had been treated for breast cancer, about 24% had problems with anxiety or depression, and about 14 percent said they had disorders associated with pregnancy. The prevalence of NTFRs in our population was lower than that of TFRs, but not negligible, and their relationship with CVDs was less close but the curves had a similar trend. The topic is much studied at the moment [25,26], but we have not found any other work concerning Italian women. Increased attention to NTRFs and specific questionnaires need to enter clinical practice.

### 4.5. This Last Point Contrasts with Reports on Lifestyle, a Secondary Endpoint of This Work

A total of 5259 women (94.08%) knew that smoking is a CVRF but 21.68% smoked; 89.92% of women knew that physical activity reduces CVR but only 13.36% exercised and 34.53% walked regularly. Fruit and vegetable consumption was also abundant (more than two portions per day) in only 30.4%. In addition, about 44% of the women stated that it was difficult to change their eating habits, about 48% their physical activity habits, and 29% their smoking habits. 

Finally, a high proportion (87.44%) of women required more information about CVR and more than 77% prefer to be informed by their doctor (cardiologist or general practitioner).

## 5. Study Limitations

The present study is one of the few European studies about CVRFs, their knowledge, and awareness, with an appreciable sample. The wide territorial distribution and the variegated cultural and socio-economic levels give a representative indication of the Italian female population. 

The first important limitation was the division of research into two steps due to the interruption caused by the COVID-19 pandemic. Some questions were introduced only in the second questionnaire and related answers referred to an inferior number of patients.

The study is based on the undocumented statements of patients, whose educational level is homogeneously distributed: it provides only indicative data. Patients often gave unlikely answers, such as the fact reported in the results that 46 women over the age of 60 declared that they were not in menopause. 

The number of unanswered responses could have been lower if the patients had been guided through the compilation instead of only receiving an initial explanation. This was a precise choice so as not to affect the results.

## 6. Conclusions

The data from the present work in line with other previous work highlighted good knowledge of CVFRs but at the same time an underestimation of one’s own CVR. In Italian women, the underestimation was greater the higher the CVR. There is a lack of adequate consideration of female CVR compared to male. Education seems to influence the number of CVRFs and the perception of one’s own risk. Healthy lifestyles are also well known but under-practiced.

A specific risk assessment considering gender-specific or gender-prevalent risk factors in women is necessary. Moreover, we believe that education in assessing one’s own CVR and leading a healthy lifestyle must begin as early as in school and must continue to be promoted by all healthcare professionals. 

## Figures and Tables

**Table 1:** Age, BMI, marital status, and educational level of the assessed population.

	Subjects	%	Median	IQR
Women	5590			
Age			56	44–65
<46	1182	21.14		
46–55	1478	26.44		
56–70	2072	37.07		
>70	793	14.19		
BMI	5361	95.90	25	22–29
<26 Normal	2847	53.11		
26–30 Overweight	1641	30.61		
>30 Obese	873	16.28		
Marital status	5488	98.18		
Married	3463	61.95		
Maiden	826	14.78		
Separated or Divorced	712	12.74		
Widows	487	8.71		
Education	4868	87.08		
Primary school	698	12.49		
Lower secondary school	1291	23.09		
Upper secondary school	1934	34.60		
University	955	17.08		

**Table 2:** Prevalence of CVRFs and CVDs in 5590 women.

CVRFs	Yes (n)	Yes (%)	No Answer (%)
Non-gender-specific RFs			
Arterial hypertension	1773	31.72	0
Diabetes mellitus,	561	10.04	0
Hypercholesterolemia	987	17.66	0
Smoking habit	1212	21.68	6.31
No smokers	3669	65.64	
Ex-smokers	256	5.58	
<5 cigarettes	330	5.90	
6–10 cigarettes	474	8.48	
11–20 cigarettes	339	6.06	
>20 cigarettes	69	1.23	
Overweight/obesity	1131	20.23	0
Autoimmune diseases	847	15.15	0.11
Breast cancer therapies	846	15.13	0.16
Gender-specific RFs			
Preterm births	268	4.79	0.09
Hypertension in pregnancy	209	3.72	0.07
Diabetes in pregnancy	159	2.84	0.05
Repeated miscarriages	432 *	14.38	0.10
CVDs			
Heart Failure	167	2.99	0.13
Myocardial Infarction	88	1.57	0.11
TIA/Stroke	97	1.74	0.13

Abbreviations: BMI: Body Mass Index; CVDs: Cardiovascular Diseases; CVRFs: Cardiovascular Risk Factors; RFs: Risk Factors; TIA: Transient Ischemic Attacks. (*) this topic was explored only in the second form (the percentage is calculated from 3004 women).

**Table 3:** Prevalence of anxiety, depression, and menopause. The items were investigated only in the second form (3004 women).

CVRFs	Yes (n)	Yes (%)	No Answer (%)
Anxiety and depression	724	24.15	0.40
Anxiety	476	15.85	
Depression	78	2.60	
Anxiety + Depression	170	5.66	
Menopause	1212	68.08	4.13
<1 year	92	3.06	
<2 years	93	3.10	
>2 years	1860	61.92	

**Table 4:** Awareness of own CVR in three groups with different numbers of TRFs. The report considered only 3004 pts (second form).

Number of CVRFs		Low CVR	Medium CVR	High CVR
No CVRF	n	617	615	79
	%	47.06	46.91	6.03
1 or 2	n	303	793	210
	%	23.20	60.72	16.08
3 or 4	n	6	55	37
	%	6.12	56.12	37.76

**Table 5:** Number of TRFs according to marital status and education.

N. of CVRFs	0	1–2	3–5
	%	%	%
Marital status			
Married	19.3	59.9	20.8
Maiden	37.4	52.8	8.4
Separated/divorced	19.5	67.0	13.5
Widows	4.0	70.7	25.3
Education			
Primary school	4.3	71.9	23.8
Middle school	17.6	64.3	18.0
High school	22.4	65.1	12.5
University	31.2	56.4	12.5

**Table 6:** Awareness of own CVR according to marital status, education, and menopause. *: *p* < 0.0001; **: *p* = 0.028 (*p*-values for statistical comparison between menopausal and not menopausal group).

	Low CVR		Medium CVR		High CVR		No Answer	
	n	%	n	%	n	%	n	%
All women	926	30.83	1463	48.70	326	10.85	289	9.62
Marital status								
Married	578	29.57	979	50.08	199	10.18	199	10.18
Maiden	178	42.69	176	42.21	33	7.91	30	7.19
Separated/divorced	79	29.59	129	48.31	40	14.98	19	7.12
Widows	68	22.90	147	49.49	49	16.50	33	11.11
Menopausal								
Yes	532 *	26.01	1028 **	50.27	260 *	12.71	225	11.00
No	353 *	42.28	382 **	45.75	53 *	6.35	47	5.63
Education								
Primary school	104	26.07	163	40.85	60	15.04	72	18.05
Middle school	206	29.99	309	44.98	86	12.52	86	12.52
High school	306	31.10	517	52.54	97	9.86	64	6.50
University	172	34.13	265	52.58	46	9.13	21	4.17

**Table 7:** Lifestyle.

Physical Activity	n	%
No answer	348	6.23
No activity	857	15.33
Housework	1708	30.55
Walk	1930	34.53
Training	747	13.36
Daily portions of fruit/vegetables		
No answer	297	5.31
0	167	2.99
1 or 2	3427	61.31
3 or 4	1324	23.69
>4	375	6.71
Smoking cigarettes		
No answer	453	8.10
Ex-smokers	256	5.58
No smokers	3669	65.64
Smokers	1212	21.68
<5 cigarettes	330	5.90
6–10 cigarettes	474	8.48
11–20 cigarettes	339	6.06
>20 cigarettes	69	1.23
Most difficult goal		
No answer	418	7.48
Correct eating habits	1649	29.50
Regular exercise	1890	33.81
No smoking	815	14.58
All previous three	818	14.63

**Figure A1:**
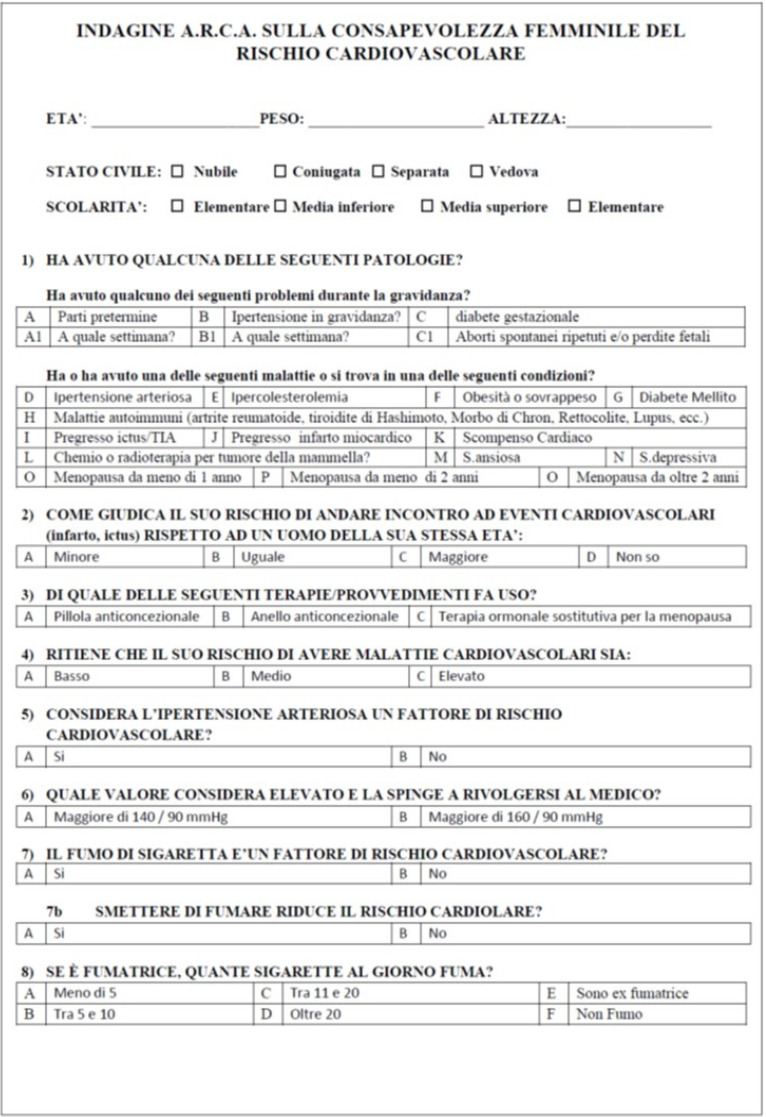

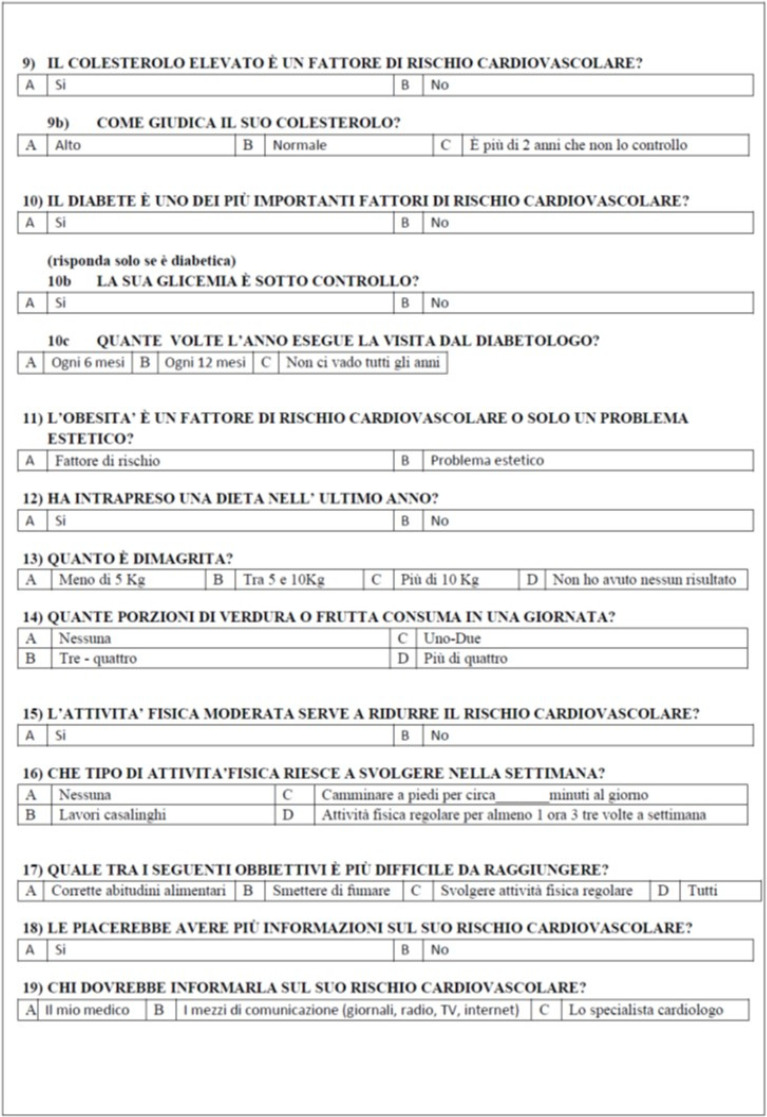
Questionnaire (second form).

## Data Availability

The data presented in this study are available on request from the corresponding author due to privacy restriction regarding researchers.
